# Correction: Immunization coverage and its associated factors among children aged 12–23 months in Ethiopia: An umbrella review of systematic review and meta-analysis studies

**DOI:** 10.1371/journal.pone.0302715

**Published:** 2024-04-22

**Authors:** Alemu Birara Zemariam, Gebremeskel Kibret Abebe, Mulat Awoke Kassa, Addis Wondemagegn Alamaw, Rediet Woldesenbet Molla, Biruk Beletew Abate, Befkad Derese Tilahun, Wubet Tazeb Wondie, Rahel Asres Shimelash, Molla Fentanew

The seventh author’s name is spelled incorrectly. The correct name is: Befkad Derese Tilahun.

## References

[1] Zemariam AB, Abebe GK, Kassa MA, Alamaw AW, Molla RW, Abate BB, et al. (2024) Immunization coverage and its associated factors among children aged 12–23 months in Ethiopia: An umbrella review of systematic review and meta-analysis studies. PLoS ONE 19(3): e0299384. 10.1371/journal.pone.0299384. 38451961 PMC10919590

