# Supplementary material for: Immunization coverage and its associated factors among children aged 12–23 months in Ethiopia: An umbrella review of systematic review and meta-analysis studies
Source: PLoS One. 2024 Mar 7;19(3):e0299384. doi: 10.1371/journal.pone.0299384 (PMC10919590; doi:10.1371/journal.pone.0299384)
Supplement: Attachment — Submitted filename: Response to reviewers.docx [file pone.0299384.s003.docx]

**November 13/2023**

**Point by point response letter**

**Subject: submission of revised manuscript and point by point response for reviewers’ comments**

**Manuscript ID**: PONE-D-23-21407

**Manuscript title**: Immunization Coverage and its Associated Factors among Children Aged 12-23 Months in Ethiopia: An Umbrella Review of Systematic Review and Meta-Analysis Studies

**To: - PLOS ONE**

**Dear respected editors and reviewers,**

We would like to thank you for these constructive, building, and improvable comments on this
manuscript that would improve the substance and content of the manuscript. We have considered each comments and clarification questions of reviewers on the manuscript thoroughly. Our
point-by-point responses for each comment and question are described in detail on the following
pages. Furthermore, the details of changes were shown by track changes using red color on the recently attached file labeled “revised manuscript with track changes” along with this we have also uploaded the detail point by point response labeled “ response to reviewers” and unmarked version of revised manuscript file labeled “manuscript”. The manuscript language was checked by language professionals and we have followed the journal guidelines upon preparation and submission of the manuscript.

Dear Dr. Zemariam,

Thank you for submitting your manuscript to PLOS ONE. After careful consideration, we feel that it has merit but does not fully meet PLOS ONE’s publication criteria as it currently stands. Therefore, we invite you to submit a revised version of the manuscript that addresses the points raised during the review process.

We look forward to receiving your revised manuscript.

Kind regards,

Tamirat Getachew

Academic Editor

PLOS ONE

Journal Requirements:

When submitting your revision, we need you to address these additional requirements.

1. Please ensure that your manuscript meets PLOS ONE's style requirements, including those for file naming. The PLOS ONE style templates can be found at

<https://journals.plos.org/plosone/s/file?id=wjVg/PLOSOne_formatting_sample_main_body.pdf> and <https://journals.plos.org/plosone/s/file?id=ba62/PLOSOne_formatting_sample_title_authors_affiliations.pdf>

**Authors’ response:** Dear many thanks for your recommendation. We have followed the journal guideline upon preparation and submission of the manuscript in order to fulfill the journal requirements.

2. Note from Emily Chenette, Editor in Chief of PLOS ONE, and Iain Hrynaszkiewicz, Director of Open Research Solutions at PLOS:  Did you know that depositing data in a repository is associated with up to a 25% citation advantage

(<https://doi.org/10.1371/journal.pone.0230416>)? If you’ve not already done so, consider depositing your raw data in a repository to ensure your work is read, appreciated and cited by the largest possible audience. You’ll also earn an Accessible Data icon on your published paper if you deposit your data in any participating repository (<https://plos.org/open-science/open-data/#accessible-data>).

**Authors’ response:** Dear respected thank you so much for your suggestion. We have tried to deposit our raw data in a repository as per your recommendation. Again thank you for your vital suggestion.

3. We noticed you have some minor occurrence of overlapping text with the following previous publication(s), which needs to be addressed: In your revision ensure you cite all your sources (including your own works).

- <http://dx.doi.org/10.1155/2019/5302307> - <http://dx.doi.org/10.34172/hpp.2022.15>

- <https://doi.org/10.1186/s12889-020-09890-0>

**Authors’ response:** Dear thank you very much for your important suggestion. We have acknowledged the author by citing these all previous published sources or articles.

4. We note that you have indicated that data from this study are available upon request. PLOS only allows data to be available upon request if there are legal or ethical restrictions on sharing data publicly. For more information on unacceptable data access restrictions, please see <http://journals.plos.org/plosone/s/data-availability#loc-unacceptable-data-access-restrictions>.

In your revised cover letter, please address the following prompts:

a) If there are ethical or legal restrictions on sharing a de-identified data set, please explain them in detail (e.g., data contain potentially sensitive information, data are owned by a third-party organization, etc.) and who has imposed them (e.g., an ethics committee). Please also provide contact information for a data access committee, ethics committee, or other institutional body to which data requests may be sent.

b) If there are no restrictions, please upload the minimal anonymized data set necessary to replicate your study findings as either Supporting Information files or to a stable, public repository and provide us with the relevant URLs, DOIs, or accession numbers. For a list of acceptable repositories, please see <http://journals.plos.org/plosone/s/data-availability#loc-recommended-repositories>. We will update your Data Availability statement on your behalf to reflect the information you provide.

**Authors’ response:** Dear we would like to ask a great excuse for the inconsistency that we made. The data set used for this study is an anonymized data set and there is no legal or ethical restriction. Thus, the authors confirm that all data underlying the findings are fully available without restriction. All the relevant data are included within the manuscript and are included in the supplementary information files. Kindly see data availability statement of the revised manuscript.

5. Please include a separate caption for each figure in your manuscript.

**Authors’ response:** Thank you very much. We have revised and corrected it accordingly. Kindly see the revised manuscript version.

6. Please include captions for your Supporting Information files at the end of your manuscript, and update any in-text citations to match accordingly. Please see our Supporting Information guidelines for more information: <http://journals.plos.org/plosone/s/supporting-information>.

**Authors’ response:** Thank you. We have accepted and added it as per your recommendation and the journal guideline.

**Additional Editor Comments:**

You need to address each and every comment and question raised by the reviewers, and additionally:-
You are expected to include the following under Table 1:

1. Search strategies used by the included SRM

2. Author conclusions

**Authors’ response**: Dear editor we would like to thank you for your vital issues you had raised. We have accepted and added it accordingly. Kindly see the revised manuscript version of table 1.

Reviewers' comments:

Reviewer's Responses to Questions

**Comments to the Author**

1. Is the manuscript technically sound, and do the data support the conclusions?

Reviewer #1: Partly

Reviewer #2: Yes

2. Has the statistical analysis been performed appropriately and rigorously?

Reviewer #1: Yes

Reviewer #2: Yes

3. Have the authors made all data underlying the findings in their manuscript fully available?

Reviewer #1: Yes

Reviewer #2: Yes

4. Is the manuscript presented in an intelligible fashion and written in standard English?

Reviewer #1: No

Reviewer #2: Yes

5. Review Comments to the Author

Please use the space provided to explain your answers to the questions above. You may also include additional comments for the author, including concerns about dual publication, research ethics, or publication ethics. (Please upload your review as an attachment if it exceeds 20,000 characters)

Reviewer #1: Comments to the authors

Abstract,

1. Line 27: In Ethiopia, there was limited, and inconclusive studies conducted so far regarding immunization coverage [grammar and readability]. In Ethiopia, limited and inconclusive studies have been conducted on immunization coverage so far. Line 31: This umbrella review was included five systematic reviews and meta-analysis studies [grammar and readability]. This umbrella review included five systematic reviews and meta-analyses.

**Authors’ response**: Dear reviewer thank you so much for your suggestion. We have accepted and revised it accordingly. Kindly see the revised manuscript of the abstract section.

2. Line 31 method: It is imperative to list all electronic databases used and to include the publication date of all included studies to ensure a thorough search strategy and updated evidence.

**Authors’ response**: Dear respected reviewer thank you for your very important issues you had raised. We have accepted and incorporated it accordingly. Kindly see the revised manuscript.

3. Line 39: The overall pooled full vaccination coverage was 57.72% (95% CI 50.17, 65.28). Do you think the difference in magnitude reported by individual systematic reviews has a potential impact on clinical practice and warrants an umbrella review?

**Authors’ response:** Dear reviewer thank you for your vital comments. The reported prevalence of immunization coverage varied from 47% to 65%, per the included individual systematic reviews and meta-analysis. Even though, systematic reviews and meta-analyses studies are really believed to indicate a high level of evidence for decision making in health initiatives, but when there are more individual reviews and inconsistent results found, it might become a tiresome for information users and policy makers for clinical practices and decision making. Thus, program planner and decision makers requires a single strong evidences about a health issues that helps them to design appropriate strategies and interventions which can be applied directly on the clinical settings for a better patient outcome. Thus, the significant variation on the magnitude of coverage across the individual reviews results or warrants different strategies in clinical practice and it should be re-analyzed in order to provide a single pooled estimate for different stakeholders to design appropriate strategies to halt the burden of vaccine preventable disease by boosting the level of immunization coverage. Moreover, umbrella reviews are used to integrate, evaluate, and synthesize the findings of related systematic reviews. By organizing and summarizing the abundant information in accordance with the level of evidence, this umbrella review can serve as a useful methodological tool and provide appropriate entry points to clinicians or decision-makers in the medical field in particular of expanded program of immunization program planners and policy makers. Considering the availability of many suitable interventions for specific conditions in a broad field, the current umbrella review can enable evidence-based decision-making and offer a broad perspective for the resolution of issues in healthcare by summarizing the evidence and providing directions on immunization coverage and its predictors in resource limited settings including Ethiopia. The current umbrella review is clearly contributing to the management of the deluge of evidence in evidence-based medicine for providing several directions for policy makers, clinicians and future researchers on the area of immunization. Therefore, this umbrella review can have a significant impact on research, policy, and practice by allowing decision-makers to abstract evidence to broader scales when assessing the strengths and limitations of current knowledge regarding immunization coverage and its predictors in Ethiopia. Additionally, kindly see the revised manuscript of the implication of the study section.

4. Considering the quality of reviews included in the umbrella review, how do you classify the review?

**Authors’ response:** Dear reviewer thank you so much for your vital issues. AMSTAR has been recommended for assessing the methodological quality of systematic reviews and meta-analyses studies among the other various tools, as it possesses good reliability in clinical settings and has undergone both internal and external validation (Nair, R., *Umbrella Reviews: Evidence Synthesis with Overviews of Reviews and Meta-Epidemiologic Studies*. 2017) and it has 11 items scored “yes,” “no,” “cannot answer,” or “not applicable.” Scores 0–4, 5–8, and 9–11 indicate low-, moderate-, and high-quality reviews (Shea, B.J., et al., *Development of AMSTAR: a measurement tool to assess the methodological quality of systematic reviews.* BMC medical research methodology, 2007. **7**(1): p. 1-7), respectively. Authors conducted the appraisal independently and it was found that ranged from 10 to 11, with a mean score of 10.6 points, indicating an overall high quality.

5. Line 48: This study showed the full immunization coverage in Ethiopia was lower compared to the WHO-recommended level and was significantly associated with a number of factors.
   Does this umbrella review summarize immunization coverage evidence, address variation in evidence, or present pooled evidence and compare it to global standards?

**Authors’ response:** Dear reviewer many thanks for your nice comment. The primary aim of this umbrella review is to summarize the heterogeneous findings of the SRM studies about immunization coverage into a single comprehensive document where the results of these reviews can be compared and contrasted in order to be utilized by clinicians and child health policy makers to design evidence-based public health responses and guide them to improve immunization coverage and to enhance the clinical practices and patient outcomes by minimizing the burden of vaccine preventable disease in the country, thereby enabling achievement of the SDG target of reducing preventable child mortality by 2030 and achieving the WHO vaccination coverage recommendation target level. But, the authors also considered the comparison of the pooled national coverage with the WHO target in order to provide an insight for the policy makers and program planners to promptly work hard with different interventions and strategies towards achieving the global recommended immunization coverage target level.

6. You mentioned several factors that affect immunization coverage. Can you elaborate on these factors and the implications of the findings for clinical practice, policy, and future research?

**Authors’ response:** Dear respected reviewer thank you so much for your critical questions you had raised. The current umbrella reviews identified several factors which have a direct implication for clinical practice, policy and future researchers by organizing and summarizing the abundant information in accordance with the level of evidence, the current umbrella review can serve as a useful methodological tool and provide appropriate entry points to clinicians or decision-makers in the immunization coverage. Therefore, policy makers shall be focused on the identified predictors by enhancing maternal knowledge regarding immunization, access to health institution which provides both vaccination, antenatal care, and delivery related services, thereby, enabling achievement of the SDG target of reducing preventable child mortality by 2030 and achieving the WHO vaccination coverage recommendation target level. Moreover, clinicians shall better to work by maximizing the immunization schedule, outreach services, home visit, and maternal awareness creation. Besides, kindly see the revised manuscript of implication of the study section since we have tried to address your issues in detail over there.

Introduction

7. Line 56: The first paragraph entails global under-five mortality; however, it doesn’t particularly show the death rate attributed to incomplete immunization, which clearly reveals the burden of the problem.

**Authors’ response**: Dear reviewer thank you for the comment. We have accepted and revised it accordingly. Kindly see the revised manuscript.

8. I suggest reordering the sequence of the paragraphs in such a way that the third paragraph is pooled before the second paragraph.

**Authors’ response**: Many thanks for your suggestion. We have accepted and re-ordered as per your recommendation. Kindly see the revised manuscript of introduction section.

9. This section does not explain why this umbrella review is needed by discussing the quality of existing reviews, their differences in scope, and the potential impact of these differences on clinical practice or policy.

**Authors’ response**: Thank you for your nice comments. We have revised it and kindly see the last paragraph of the revised manuscript introduction section.

Method

10. Line 95: The objective of this review was to combine systematic review and meta-analysis studies to get a single pooled estimate of vaccination coverage and its predictors in Ethiopia. What is the level of immunization coverage in Ethiopia, and what are the key determinants of full immunization coverage among Ethiopian children between the ages of 12 and 23 months? These are the two research questions that the researcher attempted to answer.

Avoid the last sentence.

**Authors’ response:** Thank you dear reviewer for your suggestion. We have accepted and corrected it as per your suggestion. Kindly please have look at the revised manuscript.

11. Is there any guideline that you follow for conducting and reporting this umbrella review?

**Authors’ response:** Dear reviewer thank you for your interesting comments. The key output of the current umbrella reviews is a systematic and standardized assessment of all the evidence regarding immunization coverage and its predictors based on published systematic reviews and meta-analyses. Therefore, off course we have used a standardize guideline for conducting and reporting this umbrella review and we have cited it under the methods section. Kindly please look at over there.

12. Line 101: From May 1 to 30, 2023, two authors conducted electronic searches using sources for both published and unpublished literature from PubMed, Cochrane Central, CINHALE, Medline, Web of Science, Embase, Scopus, regional university repositories, Prospero, and grey literature. It is necessary to separately state that Prospero, the International Prospective Register of Systematic Reviews, was used to identify systematic reviews that are underway on a particular topic.

**Authors’ response:** Dear reviewer thank you so much for your vital suggestion. We have accepted and revised it accordingly. Kindly see the revised manuscript.

13. What databases are used for searching gray and unpublished works, as well as the search strategy that includes both gray and peer-reviewed literature?

**Authors’ response:** Dear reviewers pardon us for the inconsistency. Initially, we have considered locally available libraries for possible identification of SRM studies. Unfortunately, we didn’t get any unpublished works and therefore, we decided to cancel it from our search strategies. Kindly see the revised manuscript search strategy section.

14. Line 118: Study Design: Only systematic reviews and meta-analysis studies that tried to synthesize quantitative or qualitative primary studies were included. What does this mean? Is a systematic review of the prevalence of immunization coverage and its determinants considered a qualitative primary study?

**Authors’ response:** Dear reviewer great excuse for the inconsistency. The authors want to say the current umbrella review considered both systematic reviews and meta-analyses for systematically synthesize of evidences and pooled estimate of immunization coverage and its predictors. There was no one study that was assessed the prevalence of immunization coverage and its determinants qualitatively since it was only assessed or addressed by a quantitative approach. We have accepted and revised in this way. Kindly see the revised manuscript.

15. The method section did not mention small study effects or excess significance bias. Have you performed any tests to check for these biases?

**Authors’ response:** Dear reviewer thank you so much for your very interesting comments. Excess significance bias can be assessed by using graphically via funnel plot and statically using p-values of begs correlation and Egger’s regression test. However, many scholars said that a minimum of ten studies is required to evaluate publication bias ( Valentine JC, Pigott TD, Rothstein HR. How many studies do you need? A primer on statistical power for meta-analysis. Journal of Educational and Behavioral Statistics. 2010;35(2):215-47); therefore, we have not assessed the publication bias of this umbrella review since it was included only five studies. But, if it is mandatory to report despite this evidence I will write and interprete the result of begs correlation and Egger’s regression test as well as funnel plot tests on the revised manuscript soon.

Result:

16. Line 136: Both quantitative and qualitative data were reviewed and extracted. What type of qualitative data is included in a systematic review of the prevalence and associated factors of immunization coverage? Do you mean the qualitative synthesis and quantitative analysis, or the meta-analysis?

**Authors’ response**: Dear reviewer thank you for your understandings and comments. As you said the author wants to say that the current umbrella review has been done by qualitative synthesis and quantitative analysis of the existing available evidences and we have only quantitative data regarding the immunization coverage its predictors that were reviewed and extracted using a standardized data abstraction form. We have revised it accordingly.

17. Line 200: The scoring system for assessing the methodological quality of the included SRMs was not explicitly presented, making it difficult to determine whether a study was of good quality.

**Authors’ response**: Thank you for your very important issues. We have addressed your issues accordingly. Kindly see the revised manuscript of methodological quality assessment sub section of the result section. Moreover, we have cited a reference and stated each of these criteria in this section.

18. The grading of evidence in an umbrella review allows for the assessment of the certainty of the overall evidence for a particular research question based on the quality and consistency of the evidence from the included systematic reviews. However, you have not included the grading of evidence for the included systematic reviews.

**Authors’ response**: Dear reviewer many thanks for the comment. We have grade and classify the quality score of the included SRM studies based on a standardized criteria. Kindly see the revised manuscript version.

Discussion:

19. Amplify your discussion by including a synthesis of the findings of systematic reviews, discussing implications, comparing to previous research, addressing strengths and limitations, and suggesting future research directions.

**Authors’ response**: Dear reviewer thank you so much for your vital suggestion. We have accepted and incorporated your issues under the revised manuscript discussion section. Kindly have a look at there.

Conclusion:
The comment is included in the abstract section.

**Authors’ response**: Dear reviewer many thanks for your vital comments. We have addressed your issues on the above authors’ response (Q-4, 5, and 6)

Reviewer #2:

1. The authors didn’t mention the vaccines that should be completed at this age in their country

This is crucial for the audience to comprehend the system of vaccination in this country.

**Authors’ response**: Dear reviewer thank you so much for your important issues. As we know that Ethiopia adopted the expanded program of immunization (EPI) from world health organization in 1974 and all the vaccines should be given as per the EPI schedules. Therefore, the schedules are listed as follows with their respected age. At birth the child should took BCG, OPV0, at 6 weeks, OPV1, Penta1, Rota 1, at 10 weeks, OPV2, Penta 2, Rota2, at 14 weeks, OPV3, Penta 3, at 9 month, MCV1, Vit A, and at 15 months, MCV2. Therefore, the children on this age group are expected to complete immunization according to EPI schedule.

2. A weird abbreviation of systematic reviews and meta-analysis SRM is written without explanation of this abbreviation.

**Authors’ response**: Dear reviewer thank you. We have explained this abbreviation on its first appearance and also included under the abbreviations section. Moreover, we had written it as a footnote when we used it under table and figures section. Kindly see over there on the revised manuscript.

3. The authors tried to magnify the importance of this pooled estimate and wrote in the introduction fragmented studies while in fact there were systematic reviews tackling the same issue. How they can call a study done on thousands of children fragmented studies. The importance of the study or the gap of knowledge is rather weak. The audience could clearly get the range of vaccination in the country from the introduction section which is very narrow to the overall estimate of the study. This amplification of the importance of the study is not justified by the authors.

**Authors’ response**: Dear reviewer thank you for your comments. We have addressed your issues explicitly on the introduction section. Kindly see the last paragraph of the revised manuscript version and under the implication of the study section.

Results

4. There are two numbers of heterogeneity score in the same section for the overall estimate of vaccination coverage. They are both high. The quality of the figure that summarized the whole results in stata output is rather very bad. I think they might want to consider getting a higher quality output graph and enlarge the fonts and numbers to give the message clearly.

**Authors’ response**: Dear reviewer excuses me for the inconsistency. The heterogeneity results was I^2^ =99.7%, P<0.001, this is the correct heterogeneity test result of the current umbrella review and we have revised the manuscript accordingly. We have uploaded a clear visualize figures or graphs. Kindly request you to see the revised graph and manuscript.

5. The title of the graph or figure 2 is not supposed to include the term umbrella review or the name of the article??

**Authors’ response**: Thank you so much. We have revised and labeled as “umbrella review about the pooled immunization coverage among children aged 12-23 months in Ethiopia”. Kindly see the revised manuscript of figure-2 title.

Limitation of the study

6. I think they should add that the heterogeneity is very high for the overall prevalence of vaccination and the figure that they had, has to be taken with caution. This is a huge limitation of the final findings of this review. The authors totally ignored this high level of heterogeneity and didn’t explain why this figure was very high

**Authors’ response**: Dear reviewer wholeheartedly thanks for your insightful view. The authors believed that the high figure of heterogeneity might be due to a small number of SRM studies included (4 studies) and a huge variation across primary studies included by the systematic reviews and meta-analyses. Therefore, the author recommends for the future researcher in order to do an umbrella review by including many more meta-analysis articles to reduce the heterogeneity and to identify many more associated factors of immunization coverage. We have accepted and included as a limitation of the current umbrella review. Kindly see the revised manuscript version.

6. PLOS authors have the option to publish the peer review history of their article ([what does this mean?](https://journals.plos.org/plosone/s/editorial-and-peer-review-process#loc-peer-review-history)). If you choose “no”, your identity will remain anonymous but your review may still be made public. **Do you want your identity to be public for this peer review?**

Reviewer #1: No

Reviewer #2: No
