# Supplementary material for: Immunization coverage and its associated factors among children aged 12–23 months in Ethiopia: An umbrella review of systematic review and meta-analysis studies
Source: PLoS One. 2024 Mar 7;19(3):e0299384. doi: 10.1371/journal.pone.0299384 (PMC10919590; doi:10.1371/journal.pone.0299384)
Supplement: Attachment — Submitted filename: Response to reviewers.docx [file pone.0299384.s004.docx]

**December 10/2023**

**Point by point response letter**

**Subject: submission of revised manuscript and responses for reviewers’ comments**

**Manuscript ID**: PONE-D-23-21407R1

**Manuscript title**: Immunization Coverage and its Associated Factors among Children Aged 12-23 Months in Ethiopia: An Umbrella Review of Systematic Review and Meta-Analysis Studies

**To: - PLOS ONE**

**Dear respected editors and reviewers,**

We would like to thank you for these constructive, building, and improvable comments on this
manuscript that would improve the substance and content of the manuscript. We have considered each comments and clarification questions of reviewers on the manuscript thoroughly. Our
point-by-point responses for each comment and question are described in detail on the following
pages. Furthermore, the details of changes were shown by track changes using red color on the recently attached file labeled “revised manuscript with track changes” along with this we have also uploaded the detail point by point response labeled “ response to reviewers” and unmarked version of revised manuscript file labeled “manuscript”. The manuscript language was checked by language professionals and we have followed the journal guidelines upon preparation and submission of the manuscript.

Dear Dr. Zemariam,

Thank you for submitting your manuscript to PLOS ONE. After careful consideration, we feel that it has merit but does not fully meet PLOS ONE’s publication criteria as it currently stands. Therefore, we invite you to submit a revised version of the manuscript that addresses the points raised during the review process.

Please include the following items when submitting your revised manuscript:

- A rebuttal letter that responds to each point raised by the academic editor and reviewer(s). You should upload this letter as a separate file labeled 'Response to Reviewers'.
- A marked-up copy of your manuscript that highlights changes made to the original version. You should upload this as a separate file labeled 'Revised Manuscript with Track Changes'.
- An unmarked version of your revised paper without tracked changes. You should upload this as a separate file labeled 'Manuscript'.

If you would like to make changes to your financial disclosure, please include your updated statement in your cover letter. Guidelines for resubmitting your figure files are available below the reviewer comments at the end of this letter.

We look forward to receiving your revised manuscript.

Kind regards,

Tamirat Getachew

Academic Editor

PLOS ONE

**Authors’ response**: Dear respected editor I would like to thank you from the bottom of my heart for your invaluable efforts made on this manuscript with a timely update and response. We have included all the above requested files separately. Kindly take a look at there.

Journal Requirements:

Please review your reference list to ensure that it is complete and correct. If you have cited papers that have been retracted, please include the rationale for doing so in the manuscript text, or remove these references and replace them with relevant current references. Any changes to the reference list should be mentioned in the rebuttal letter that accompanies your revised manuscript. If you need to cite a retracted article, indicate the article’s retracted status in the References list and also include a citation and full reference for the retraction notice.

**Authors’ response**: Dear thank you for remembering us. We have not included the extracted articles in our reference list. However, we have added the accessed date on reference number 8 and 67. Kindly see the revised section of the reference list.

Reviewers' comments:

Reviewer's Responses to Questions

**Comments to the Author**

1. If the authors have adequately addressed your comments raised in a previous round of review and you feel that this manuscript is now acceptable for publication, you may indicate that here to bypass the “Comments to the Author” section, enter your conflict of interest statement in the “Confidential to Editor” section, and submit your "Accept" recommendation.

Reviewer #1: All comments have been addressed

2. Is the manuscript technically sounds, and do the data support the conclusions?

Reviewer #1: Yes

3. Has the statistical analysis been performed appropriately and rigorously?

Reviewer #1: Yes

4. Have the authors made all data underlying the findings in their manuscript fully available?

Reviewer #1: Yes

5. Is the manuscript presented in an intelligible fashion and written in Standard English?

Reviewer #1: No

6. Review Comments to the Author

Please use the space provided to explain your answers to the questions above. You may also include additional comments for the author, including concerns about dual publication, research ethics, or publication ethics. (Please upload your review as an attachment if it exceeds 20,000 characters)

Reviewer #1: Comments to authors

I would like to thank the corresponding author for the concise and reasonable response to the questions.
Abstract:
Line 34: Only systematic reviews and meta-analyses from inception to 1 May 2023 and restricted to English language document were included. If it makes sense, you could improve your sentence as follows. Only systematic reviews and meta-analyses published in English from inception to May 1, 2023, were included.

**Authors’ response**: Dear reviewer thank you for your vital suggestion. We have revised and corrected it accordingly. Kindly see the revised manuscript.

Line 49: This study showed the full immunization coverage in Ethiopia was lower compared to the WHO-recommended level and it was significantly associated with a number of factors.
What are those factors identified in this umbrella review? with a more direct and concise statement.
This umbrella review identifies several factors that contribute to higher immunization coverage. These factors include:

**Authors’ response**: Dear respected reviewer thank you for your important issue you had raised. We have accepted and re-write based on your vital suggestion. Kindly see the revised version of the manuscript in the abstract conclusion section.

Introduction
Line 66: Immunization estimated to prevent nearly 2 to 3 million deaths every year from vaccine-preventable diseases like diphtheria, tetanus, pertussis, influenza, and measles. Consider improving the sentence as follows: Child immunization is estimated to avert nearly 2 to 3 million deaths annually from vaccine-preventable diseases such as diphtheria, tetanus, pertussis, influenza, and measles.

**Authors’ response**: Dear reviewer thank you for your nice suggestion. We have accepted and corrected it accordingly. Kindly see the revised introduction section of the second paragraph.

Information Sources and Search Strategy

Line 127: Abbreviation: please try to put all abbreviations in full on their first appearance. For instance, MCV2.

**Authors’ response**: Dear reviewer we would like to extend our deepest gratitude to you for your critical view, important comments and suggestions. We have accepted and corrected it.

Method and material

Line 106: Based on the methodological approach of the umbrella review [20], a systematic synthesis of the eligible SRM studies on immunization coverage and its predictors among children aged 12-23 months in Ethiopia was conducted. The sentence could benefit from clearer and more consistent language. Would you consider rephrasing it?

**Authors’ response**: Thank you so much dear reviewer. We have accepted and revised it accordingly.
Screening and select

Line 153: Data on immunization coverage and its predictors were systematically reviewed and extracted using a standardized data abstraction form developed in Microsoft Excel.

**Authors’ response**: Dear reviewer thanks you so much. We have revised and corrected it accordingly. Kindly see the revised manuscript.

7. PLOS authors have the option to publish the peer review history of their article ([what does this mean?](https://journals.plos.org/plosone/s/editorial-and-peer-review-process#loc-peer-review-history)). If published, this will include your full peer review and any attached files.
If you choose “no”, your identity will remain anonymous but your review may still be made public.
**Do you want your identity to be public for this peer review?** For information about this choice, including consent withdrawal, please see our [Privacy Policy](https://www.plos.org/privacy-policy).

Reviewer #1: No
