# Supplementary material for: Immunization coverage and its associated factors among children aged 12–23 months in Ethiopia: An umbrella review of systematic review and meta-analysis studies
Source: PLoS One. 2024 Mar 7;19(3):e0299384. doi: 10.1371/journal.pone.0299384 (PMC10919590; doi:10.1371/journal.pone.0299384)
Supplement: Attachment — Submitted filename: Response to Reviewers.docx [file pone.0299384.s005.docx]

**Jan 31/2023**

**Point by point response letter**

**Subject: submission of revised manuscript and point by point response**

**Manuscript ID**: PONE-D-23-21407R2

**Manuscript title**: Immunization Coverage and its Associated Factors among Children Aged 12-23 Months in Ethiopia: An Umbrella Review of Systematic Review and Meta-Analysis Studies

**To: - PLOS ONE**

**Dear respected editors and reviewers,**

We would like to thank you for these constructive, building, and improvable comments on this
manuscript that would improve the substance and content of the manuscript. We have considered each comments and clarification questions of reviewers on the manuscript thoroughly. Our
point-by-point responses for each comment and question are described in detail on the following
pages. Furthermore, the details of changes were shown by track changes using red color on the recently attached file labeled “revised manuscript with track changes” along with this we have also uploaded the detail point by point response labeled “ response to reviewers” and unmarked version of revised manuscript file labeled “manuscript”. The manuscript language was checked by language professionals and we have followed the journal guidelines upon preparation and submission of the manuscript.

Dear Dr. Zemariam,

Thank you for submitting your manuscript to PLOS ONE. After careful consideration, we invite you to submit a revised version of the manuscript that addresses the points raised during the review process.

**ACADEMIC EDITOR:**

- Please try to address comments accordingly to avoid repeated revisions, as this is too time-consuming.

Kind regards,

Tamirat Getachew

Academic Editor

Journal Requirements:

Please review your reference list to ensure that it is complete and correct. If you have cited papers that have been retracted, please include the rationale for doing so in the manuscript text, or remove these references and replace them with relevant current references. Any changes to the reference list should be mentioned in the rebuttal letter that accompanies your revised manuscript. If you need to cite a retracted article, indicate the article’s retracted status in the References list and also include a citation and full reference for the retraction notice.
**Authors’ response**: Dear Academic Editor, thank you sincerely for dedicating your time to review our manuscript. We greatly appreciate your consideration and the kind words you have shared. We have taken into account each of the reviewers' comments and the requirements set by the journal. We have carefully revised the manuscript accordingly. Please find attached the revised version for your kind perusal. Thank you once again for your valuable input and guidance.
Reviewers' comments:

Reviewer's Responses to Questions

**Comments to the Author**

1. If the authors have adequately addressed your comments raised in a previous round of review and you feel that this manuscript is now acceptable for publication, you may indicate that here to bypass the “Comments to the Author” section, enter your conflict of interest statement in the “Confidential to Editor” section, and submit your "Accept" recommendation.

Reviewer #1: All comments have been addressed

2. Is the manuscript technically sounds, and do the data support the conclusions?

Reviewer #1: Yes

3. Has the statistical analysis been performed appropriately and rigorously?

Reviewer #1: Yes

4. Have the authors made all data underlying the findings in their manuscript fully available?
Reviewer #1: Yes

5. Is the manuscript presented in an intelligible fashion and written in standard English?

Reviewer #1: Yes

6. Review Comments to the Author

Please use the space provided to explain your answers to the questions above. You may also include additional comments for the author, including concerns about dual publication, research ethics, or publication ethics. (Please upload your review as an attachment if it exceeds 20,000 characters)

Reviewer #1: A). Use bullet points strategically to preserve the text's natural flow and formality.
**Authors’ response**: Dear respected reviewer thank you for your suggestion. We have made a revision based on your concrete suggestion. Kindly see the revised manuscript.

B). Please reference all numbered tables in the text. Currently, numbered tables (3) in the manuscript have not been cited in the text.

**Authors’ response**: Dear reviewer, we sincerely apologize for the inconsistency and oversight in failing to cite the table. We have now rectified this issue and included the proper citation. Thank you very much for bringing it to our attention. We greatly appreciate your valuable feedback and guidance throughout the review process

C). Maintain consistent formatting while citing tables and figures in the text.

**Authors’ response**: We have taken it into account and made the necessary revisions to ensure consistency in the document. We kindly request you to review the revised version and provide your feedback. We appreciate your time and attention to our manuscript.

7. PLOS authors have the option to publish the peer review history of their article
**Do you want your identity to be public for this peer review?** For information about this choice, including consent withdrawal, please see our [Privacy Policy](https://www.plos.org/privacy-policy).

Reviewer #1: No

While revising your submission, please upload your figure files to the Preflight Analysis and Conversion Engine (PACE) digital diagnostic tool, <https://pacev2.apexcovantage.com/>. PACE helps ensure that figures meet PLOS requirements.

**Authors’ response**: Thank you for your valuable suggestion. We have uploaded the figures and carefully reviewed it to ensure that it meets all the requirements set by the journal. We are pleased to report that it fulfills the necessary criteria. We appreciate your input and guidance throughout the process. Thank you once again for your significant contribution and vital suggestions
